# Progesterone-associated adjustments in brain structure during menstruation and the periovulatory phase—an MRI study

**DOI:** 10.1016/j.ebiom.2026.106184

**Published:** 2026-02-20

**Authors:** Susanne Nehls, Elena Losse, Maya Armin, Ute Habel, Natalia Chechko

**Affiliations:** a Department of Psychiatry, Psychotherapy and Psychosomatics, Medical Faculty, RWTH Aachen, Aachen, Germany; b Institute of Neuroscience and Medicine, Brain and Behavior (INM-7), Research Centre Jülich, Jülich, Germany; c Institute of Neuroscience and Medicine: JARA-Institute Brain Structure Function Relationship (INM-10), Research Centre Jülich, Jülich, Germany

**Keywords:** Menstrual cycle, Brain imaging, Brain morphology, Progesterone, Oestradiol

## Abstract

**Background:**

Fluctuations in oestrogen (E2) and progesterone (P4) shape brain morphology across menstrual cycles. Yet, it remains unclear how these hormonal dynamics translate into region-specific changes in grey matter structure.

**Methods:**

Using structural magnetic resonance imaging (MRI), we assessed grey matter volume (GMV) and cortical thickness (CT) in 32 healthy women during the periovulatory phase (peak E2) and menstruation (low E2 and P4). Region-of-interest (ROI) and exploratory whole-brain analyses were performed, complemented by multiple regression models to assess associations between E2, P4, and brain morphology. Further, spatial colocalisation analyses were applied to link observed macrostructural changes with receptor density maps.

**Findings:**

Predefined ROI analyses targeting the amygdala and the hippocampus yielded no significant effects after correction for multiple comparisons. While E2 correlated positively with parietal regions in the periovulatory phase, P4 exhibited robust phase-dependent associations with the cerebellar and fusiform volumes in the periovulatory phase, and with the frontal volumes and widespread CT during menstruation. Spatial colocalisation with hormone receptor distributions indicated stronger structural changes in regions with higher P4 receptor density.

**Interpretation:**

These results identify P4 as a key modulator of brain morphology, with its effects varying according to the menstrual phase. Understanding the hormone-driven brain dynamics is essential for a more accurate model of female brain function and mental health.

**Funding:**

German Research Foundation.

**Research in context**

**Evidence before this study**

We searched PubMed and Web of Science from Jan 1, 2000, to Dec 31, 2024, without language restrictions, using the terms “menstrual cycle”, “oestradiol”, “progesterone”, “brain structure”, “grey matter volume”, and “cortical thickness”. Studies were included if they reported MRI-based assessments of grey matter changes across menstrual phases in healthy women. Most evidence came from studies that used small samples, region-of-interest analyses, often focusing on the amygdala or the hippocampus. The reported findings were heterogeneous, with some studies suggesting cycle-dependent structural variation but limited mechanistic insights. No pooled estimates were available due to methodological heterogeneity.

**Added value of this study**

Our study combined volumetric and surface-based morphometry with hormone assays, multiple regression models, and receptor density mapping to examine brain morphology across menstrual phases. We demonstrate that progesterone, rather than oestradiol, emerges as the stronger and more consistent modulator of grey matter volume and cortical thickness. Moreover, our spatial colocalisation analyses provide evidence that macrostructural changes preferentially occur in regions with high progesterone receptor density, offering a receptor-based mechanism for hormone-driven neuroplasticity.

**Implications of all the available evidence**

Together with existing literature, our findings suggest that dynamic, hormone-related brain reorganisation is a reproducible feature of the menstrual cycle, with progesterone playing a central role. These results refine current models of female brain plasticity and highlight critical periods of hormonal sensitivity that may contribute to increased risk for mood and anxiety disorders. Future research should explore longitudinal trajectories across different reproductive states, integrate functional and molecular imaging, and test how these brain changes link to cognitive and affective outcomes.

## Introduction

Female sex hormones play a critical role in shaping brain structure across multiple transition periods, emphasising the influence of hormonal changes on the brain over time. The menstrual cycle, governed by fluctuations in oestradiol (E2) and progesterone (P4), presents a unique model to investigate the brain's sensitivity to hormonal variation in healthy females. E2 rises during the follicular phase, peaks midcycle and before ovulation, then sharply drops, while P4 remains low until after ovulation, reaching a mid-luteal peak before falling towards menstruation. Consequently, menstruation is marked by minimal hormone levels, while ovulation reflects their dynamic peak.

Given the widespread distribution of E2 and P4 receptors across the brain, the hormonal shifts directly affect brain structure and function.^1^^,^^2^ Animal research and human post-mortem studies have shown the highest receptor concentration in the amygdala, the hippocampus, the thalamus and hypothalamus, and the cerebellum.^1^^,^^2^ Beyond reproduction, E2 and P4 are implicated in mood regulation and cognition, linking hormone dynamics to female mental health.^3^ Regions rich in sex hormone receptors, such as the amygdala and the hippocampus, also subserve emotional and cognitive processing, positioning them as critical nodes in the neurobiology of mood disorders, including premenstrual mood disorders.^4^

Emerging evidence supports the idea that naturally cycling women exhibit dynamic neuroplasticity in response to hormonal shifts. Structural MRI studies indicate cycle-related changes in grey matter volume (GMV), particularly in the hippocampus and the amygdala,^5^^,^^6^ both highly sensitive to E2 and P4. However, most research has compared the follicular and luteal phases,^7^, ^8^, ^9^, ^10^, ^11^ leaving the periovulatory phase, marked by peak E2 and rising P4, underexplored.

Hagemann et al.^12^ examined seven women with ultrasound-confirmed ovulation, and reported increased global brain volume alongside reduced cerebrospinal fluid (CSF) during ovulation compared to menstruation. In a larger sample of 38 women with LH-confirmed ovulation, De Bondt et al.^13^ identified regional changes in the insula, linking P4 to cerebellar and global volume variations across the cycle. Rising E2 levels have also been associated with morphometric markers such as brain age, as shown in a study of eight women with ultrasound-verified ovulation.^14^ These findings underscore the relevance of sex hormones in shaping brain morphology, aging, and disease risk. For example, E2 has been found to correlate with greater volume in the parahippocampal and middle frontal gyri,^15^^,^^16^ regions tied to memory and executive function, and vulnerability to Alzheimer's. Similarly, P4 has been linked to fluctuations in CSF and GMV across the cycle.^12^

Despite these advances, direct comparisons between the periovulatory phase and menstruation (i.e. early follicular phase), the two most hormonally distinct phases, remain scarce. Understanding how brain morphology responds to these extremes is crucial for a comprehensive model of the healthy female brain. This study addresses this by investigating the brain's structural changes across the periovulatory phase (POV) and menstruation (ME). We hypothesised that GMV would be significantly increased during POV compared to ME, particularly in hormone-sensitive regions such as the amygdala and the hippocampus, where changes are most consistently reported^5^^,^^6^ (Hypothesis 1a). Similarly, we expected individual differences in E2 and P4 levels to correlate with GMV in these regions (Hypothesis 2a). To expand on these, we also conducted exploratory whole-brain analyses to examine cycle effects beyond the predefined ROIs (Hypothesis 1b, 2b). To probe biological mechanisms, we used spatial colocalisation analyses combining hormone levels with brain receptor maps, testing whether GMV changes aligned with regions rich in sex hormones and GABA-A and glutamate receptors^17^^,^^18^ (Hypothesis 3). Using this approach, we have recently linked early postpartum brain recovery processes to progesterone and GABA-A receptor distribution.^19^ Finally, given the sparse and inconsistent evidence regarding cortical thickness (CT) variation across the cycle, we also explored CT differences between POV and ME and their correlation with sex hormones (Hypothesis 4).

## Methods

By means of flyers and social media, we recruited 46 cisgender women with regular menstrual cycles, no hormonal contraception for at least 6 months, normal BMI, and no psychiatric disorders. The participants were drawn from a larger study examining the neural processing of body odour from fertile women in single male and female participants to assess attraction and competition. The exclusion criteria included medication/drug use, smoking, anosmia, non-cisgender identity, non-heterosexual orientation, and being in a relationship.

### Ethic statement

All participants provided written informed consent. In accordance with the Declaration of Helsinki, the study was approved by the RWTH Aachen University ethics committee (EK 512-20) and preregistered (DRKS00029175).

### Procedure

Randomly assigned to begin MRI either during ME or POV, the participants contacted the study personnel on the first day of menstruation. Those beginning during the menstrual phase (ME) completed an MRI within the first 2 days of menstruation. Five days into menstruation (or later, when the menstrual cycle length was longer than 21 days), all participants used the Clearblue Digital Ovulation Test each morning until an oestradiol rise followed by a luteinising hormone (LH) surge was detected, supported by the Clearblue cycle calculator. The oestradiol increase signalled the approaching fertile window, while the LH surge reliably marked the two most fertile days and the timing of ovulation,^20^, ^21^, ^22^ with urinary LH tests showing a specificity of 1.00 and an accuracy of 0.97 for ovulation detection.^23^ Upon LH detection, participants scheduled the MRI measurement within the 2 fertile days. In 3 women, no LH surge was detected, indicating a possible anovulatory cycle. These participants repeated ovulation testing in the subsequent cycle.

On both measurement days (ME and POV), blood samples were taken for hormone validation (E2, P4) and the participants were asked to complete the Beck's depression Inventory (BDI II) and the Premenstrual Tension Syndrome Scale (PTSS). BDI scores were missing for five participants during menstruation and for two during POV. PTSS scores were missing for three participants during both measurements. Anatomical analyses included 32 participants (14 excluded for missing time points).

### Hormonal assays

E2 and P4 serum concentrations were measured before each scanning session and analysed by competitive immunometry electrochemistry luminescence detection at the Laboratory Diagnostic Center, University Hospital Aachen. The samples were run on a Roche Cobas e601 and on a Roche Cobas e801 with Cobas Elecsys E2 and P4 reagent kits, respectively (Roche Diagnostics, Bromma, Sweden). For P4, the measurement interval was 0.05–60 ng/ml with an intra-assay coefficient of variation (CV) of 2.33–2.49%. For E2, the measurement interval was 5–3000 pg/ml with a CV of 1.77–2.91%. E2 and P4 levels were missing for one participant during the menstruation phase.

### Behavioural data analyses

The analysis was conducted using SPSS® 29 IBM Corporation, Armonk NY, USA for Windows®. Due to positively skewed distribution of P4 and E2 levels, natural logarithmic transformation was performed. Dependent t-tests were used to calculate changes in hormonal levels between the two time points.

### MRI data acquisition and analysis

Neuroimaging data were acquired using a 3 T Prisma MR Scanner (Siemens Medical Systems, Erlangen, Germany) located in the Medical Faculty of RWTH Aachen University. T1-weighted structural images were acquired by means of a 3-dimensional magnetisation-prepared rapid acquisition gradient echo imaging sequence (4.12 min; 176 slices, TR = 2300 ms, TE = 1.99 ms, TI = 900 ms, FoV = 256 × 256 mm^2^, flip angle = 9°, voxel resolution = 1 × 1 × 1mm^3^). All images were inspected for structural abnormalities, and scanner and motion artefacts. In case of the latter two, imaging acquisition was repeated.

### Preprocessing and analysis of anatomical data

Anatomical imaging data were preprocessed using the Computational Anatomy Toolbox (CAT12.9 Version r2577) and statistical parametric mapping (SPM)12 toolbox implemented in Matlab 2022b (MathWorks, Inc., Natick, MA). For volume-based morphometry (VBM), default settings of the longitudinal protocol were applied for spatial registration, segmentation, and normalisation.^24^ All images were affine registered to standard tissue probability maps, by correcting individual head positions and orientations, and translated into Montreal Neurologic Institute (MNI) space. The images were segmented into grey matter (GM), white matter (WM), and cerebrospinal fluid. Images were visually inspected for potential segmentation and registration errors. Following the suggestions of the CAT12 toolbox manual,^24^ a homogeneity check of the unsmoothed data identified no outliers, thus the GMVs of all participants were included in subsequent analyses. Finally, the modulated GMV was smoothed with an 8-mm full-width at half-maximum (FWHM) Gaussian kernel.

For surface-based morphometry (SBM), information on cortical thickness was extracted during the longitudinal protocol for VBM. Local maxima were projected to the grey matter voxels by using neighbour relationship described by the WM distance, equaling cortical thickness. The estimation of cortical thickness was performed according to projection-based thickness including partial volume correction, sulcal blurring, and sulcal asymmetries without sulcus reconstruction.^25^ Topological correction was performed through an approach based on spherical harmonics. For interparticipant analysis, an algorithm for spherical mapping of the cortical surface was included.^26^ An adapted volume-based diffeomorphic DARTEL algorithm was then applied to the surface for spherical registration. All scans were resampled and smoothed with a Gaussian kernel of 15-mm FWHM.

### Region-of-interest (ROI) extraction and analyses

Primary analyses focused on the bilateral amygdala and hippocampus, given their high density of sex hormone receptors and consistent involvement in hormone-related brain changes.^5^^,^^6^^,^^27^ Mean GMV was extracted from these ROIs (Neuromorphometric atlas). Whole-brain GMV and CT metrics were also computed for exploratory analyses (136 GMV and 68 CT ROIs). All extracted values were then imported into SPSS® 29 for statistical analysis.

### Statistics

The brain metrics met the assumption of normal distribution (Saphiro-Wilk test). Paired t-tests assessed differences in total GMV, WMV, CSF, CT, and ROI volumes between ME and POV. Pearson partial correlations tested associations between log-transformed E2 or P4 levels and ROI/whole-brain GMV or CT. For GMV analyses, both age and total intracranial volume (TIV) were used as nuisance variables. For CT models, TIV was not included as a covariate, following CAT12 methodological recommendations indicating that CT is already normalised for head size and that including TIV may introduce statistical artefacts.^24^ The effect sizes of the significant results are reported Cohen's d for pairwise comparisons (small: 0.20–0.49, medium: 0.50–0.79, large: 0.80 and greater).^28^ Multiple regression analyses of voxel-wise GMV and CT data were conducted in CAT12, including age (CT) or age and TIV (GMV) as nuisance variables.^24^ Significance was determined using false-discovery rate (FDR) correction unless otherwise noted.

### Spatial colocalisation between VBM data and receptor distributions

To probe the neurochemical basis of structural changes, spatial colocalisation analyses were performed with JuSpace.^17^ Cycle-related GMV differences (Cohen's d) were correlated with receptor density maps for E2 (ESR1), P4 (PGR), GABA-A, and glutamate (mGluR5), derived from the Allen Human Brain Atlas.^29^ Spearman correlations were computed region-wise across the Neuromorphometric atlas, with significance assessed using 1000 spatial permutations and FDR correction.

### Role of funders

Funders had no role in study design, data collection, data analyses, interpretation, or writing of the manuscript.

## Results

Participants had a mean age of 22.91 years (SD = 3.42) and an average body-mass-index (BMI) of 23.37 (SD = 2.70). The average cycle length was 29.03 days (SD = 2.01, range = 24–33). MRI sessions were conducted on day 1.6 (SD = 0.8) for ME and day 14.5 (SD = 2.8) for POV.

Dependent t-tests confirmed higher log-transformed E2 and P4 levels during POV than ME (E2: t (30) = 11.25, p < 0.001; P4: t (30) = −4.05, p < 0.001).

Although not clinically elevated, significantly higher scores during ME compared to POV were observed for BDI (t (24) = 2.22, p = 0.018) and PTSS (t (27) = 4.67, p < 0.001). Neither score correlated significantly with hormone levels (Table 1).

**Table 1:** Means and standard deviation (SD) for raw values of oestradiol and progesterone during menstruation (ME) and the periovulatory phase (POV) and scores of the Beck's Depression Inventory (BDI) and Premenstrual Tension Syndrome Scale (PTSS).

	Mean (SD)
Oestradiol during ME (pg/ml)	30.30 (12.38)
Oestradiol during POV (pg/ml)	131.24 (60.94)
Progesterone during ME (ng/ml)	0.42 (0.26)
Progesterone during POV (ng/ml)	1.78 (2.86)
BDI during ME	2.7 (3.12)
BDI during POV	2.04 (2.61)
PTSS during ME	6.31 (5.27)
PTSS during POV	1.96 (2.63)

### Hypothesis-driven ROI-analyses

Analyses of the bilateral amygdala and hippocampus revealed no significant GMV differences between POV and ME after FDR correction. Exploratory analyses that did not survive multiple-comparison correction are reported in Supplementary Material.

No significant correlations were found between E2 or P4 and GMV in these ROIs in either phase.

### Exploratory whole-brain analyses

Total GMV was significantly higher during POV compared to ME (t (31) = 0.25, p = 0.004, d = 0.51). No differences emerged for white matter (WM), cerebrospinal fluid (CSF), CT, or total intracranial volume (TIV).

ROI analyses across 136 GMV regions revealed no significant volume increases during POV after FDR correction. Exploratory, uncorrected results of the ROI analyses are reported in Supplementary Material.

Partial correlation analyses revealed positive correlations between log-transformed P4 and total CT during POV (r = 0.522, FDR-corrected p = 0.012) and ME (r = 0.611, FDR-corrected p < 0.001). No significant correlations were observed with E2.

**Fig. 1:**
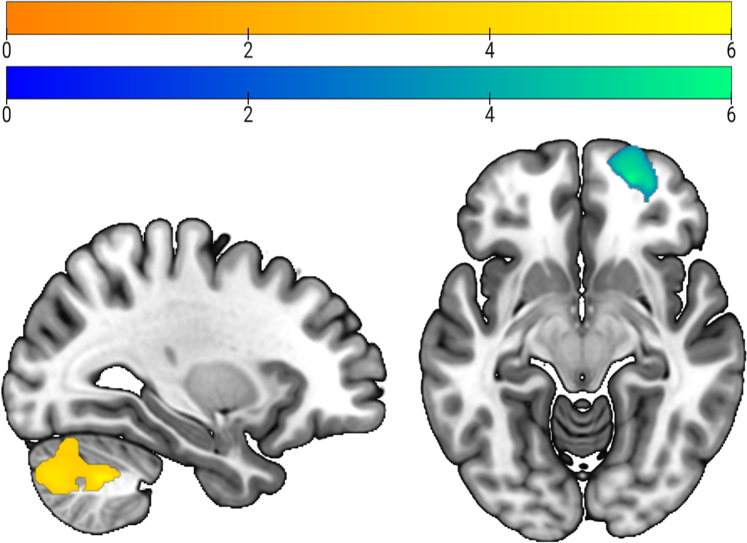
Positive correlation between progesterone levels and grey matter volume: during the perivoulatory phase, a significant effect was observed in the cerebellum (yellow). During menstruation, a significant effect appeared in the orbitofrontal gyrus (blue-green).

P4 was positively correlated with GMV in bilateral cerebellum crus 1, 2, and 6 during POV (left: 2522 voxels, peak MNI −28, −58, −39; T = 6.33; FDR-corrected p < 0.001; right 3231 voxels, right: peak MNI 9/−88, −45; T = 5.08; FDR-corrected p < 0.001). During ME, P4 positively correlated with the right superior and middle orbital, middle frontal, anterior orbital, and rectal gyri (1243 voxels, peak MNI 21, 54, −10; T = 6.12; FDR-corrected p = 0.007) (Fig. 1). Further exploratory, uncorrected correlational analyses of log-transformed E2 and P4 and GMV are reported in the Supplementary Material.

### Cortical thickness variation

Exploratory ROI analysis revealed non-corrected increased CT during POV in the right frontal pole and left fusiform gyrus.

**Fig. 2:**
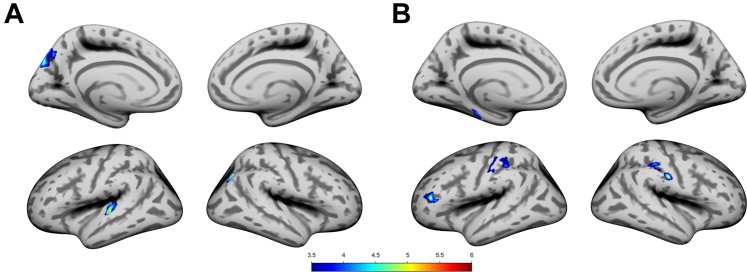
Significant FDR corrected positive correlations between progesterone levels and cortical thickness during A) the periovulatory phase and B) menstruation.

FDR-corrected partial correlation analyses showed that P4 during POV was positively correlated with CT in the left precuneus, the superior parietal lobule, the cuneus, the superior temporal gyrus and the transverse temporal gyrus, as well as with the right inferior parietal lobule (see Table 2, Fig. 2A). During ME, P4 was positively correlated with the CT of the bilateral postcentral gyrus and the supramarginal gyrus, and with the left rostral middle frontal, and the inferior frontal gyri, the p. triangularis, and the parahippocampal and fusiform gyri (see Table 2, Fig. 2B).

**Table 2:** Significant positive correlation of log-transformed progesterone with the cortical thickness across the menstrual cycle.

Overlap of atlas region	Side	T value	Cluster-size
**Menstruation**			
Postcentral (75%), supramarginal (25%)	L	4.14	138
Postcentral (100%)	L	3.86	104
Rostral middle frontal (72%), pars triangularis (28%)	L	5.05	103
Parahippocampal (76%), fusiform (24%)	L	4.81	76
Supramarginal (87%), postcentral (13%)	R	4.25	135
Postcentral (57%), supramarginal (43%)	R	5.12	82
**Periovulatory phase**			
Precuneus (44%), superior parietal (43%), cuneus (12%)	L	4.82	212
Superior temporal (52%), transverse temporal (48%)	L	5.37	161
Inferior parietal (100%)	R	5.24	76

Atlas labelling was performed according to the Desikan-Killiany atlas 28.

No correlations were observed with E2, PMS or BDI scores.

### Spatial colocalisation

Spatial colocalisation analyses revealed that the volume change from ME to POV showed significant spatial alignment with progesterone receptors (p-FDR = 0.016, spearman rho = 0.284). No significant association with E2, GABA-A and mGluR5 was found.

## Discussion

The study examined the structural changes in the female brain across the menstrual cycle with a focus on GMV and CT, and how these changes may be modulated by endogenous fluctuations in sex steroid hormones. Comparing the ovulatory and menstrual phases, we provide converging evidence that progesterone is the primary driver of macrostructural variation across the cycle.

We observed an increase in total GMV during the periovulatory phase, consistent with earlier reports of volumetric expansion during high-hormone phases.^12^ Hypothesis-driven ROI analyses of the amygdala or the hippocampus did not survive FDR-correction for multiple comparisons, although uncorrected effects (reported in the Supplementary Material) suggested periovulatory-related increases in regions related to high-level visual/face processing, semantic processing and social-emotional cognition, motor control, and cognitive function. As the findings did not survive multiple-comparison correction, they are presented solely as hypothesis-generating. While requiring cautious interpretation, their anatomical relevance however aligns with prior evidence that sex hormones exert spatially selective influences,^1^ given that these regions have consistently been found to exhibit volume variability across the menstrual cycle.^5^^,^^6^

Contrary to our second hypothesis, we found no significant association of E2 or P4 levels with amygdala or hippocampal volume when controlling for multiple comparisons. Instead, P4 emerged as the more robust modulator of both GMV and CT in other regions, suggesting its central role in hormone-driven neuroplastic adaptation. In line with prior findings, during the periovulatory phase, P4 levels were seen to positively correlate with GMV in the cerebellum and the fusiform gyrus.^7^^,^^13^ During menstruation, P4 was positively associated with GMV in the orbitofrontal cortex, a region central to emotion processing, and social decision-making.^30^ Spatial colocalisation analysis further supported a receptor-based mechanism, showing that structural changes preferentially occurred in the P4 receptor-dense regions. This pattern is consistent with histological and molecular evidence showing that P4 and its neuroactive metabolites act on specific receptor-rich circuits to shape structural plasticity, although it must be considered that the Allen Human Brain Atlas consists predominantly of male donors, potentially limiting receptor-specific inference.

In addition, the surface-based analysis revealed widespread, phase-dependent effects of P4 on CT. During the periovulatory phase, higher P4 levels were linked to increased CT in the left precuneus, the superior parietal lobule, the cuneus, and the superior and transverse temporal gyri, as well as the right inferior parietal lobule. During menstruation, correlations shifted to the postcentral, supramarginal, frontal, parahippocampal and fusiform gyri. This spatial shift suggests a dynamic and region-specific reorganisation of cortical architecture in concert with hormonal fluctuations, potentially reflecting shifting cognitive and emotional demands across the cycle. Many of these regions are functionally involved in emotion regulation, language and social communication, and memory processing, consistent with previously documented effects of P4 on mood, cognition, and sensory processing.^3^^,^^11^^,^^31^ Notably, P4 and its neuroactive metabolite allopregnanolone have been strongly implicated in reproductive-related affective disturbances, including PMS, PMDD, and postpartum depression.^32^^,^^33^ The brain regions that are central to mood disorders, such as the amygdala, the hippocampus, and the prefrontal cortex, are known to express high densities of P4 receptors, underscoring a mechanistic link between hormonal fluctuations, neuroplasticity, and mood vulnerability. This aligns with epidemiological evidence showing that peaks in female mood disorders coincide with major hormonal transitions such as puberty, menstruation, the postpartum period and perimenopause.^4^^,^^34^^,^^35^

In this regard, the pattern of P4-related neuroplasticity extends across major reproductive transition periods. For instance, adolescents show substantial structural alterations during puberty in regions involved in affective regulation and social cognition.^36^ Postpartum studies report that GMV trajectories of the first few postpartum months covary with gradual changes in circulating P4 levels,^37^ with spatial colocalisation analyses showing an overlap with brain regions rich in P4 and GABA-A receptors.^19^ The converging findings underscore the role of P4 as a key neuromodulator of plasticity, with effects that are both anatomically selective and receptor specific.

One complexity lies in the contrasting patterns of GMV trajectories observed in different reproductive states. In the menstrual cycle, GMV peaks during the periovulatory phase alongside the rises in hormone levels. By contrast, during pregnancy, both hormones reach their highest levels, while GMV progressively declines.^38^ Following the sharp drop in hormone levels after parturition, GMV is found to increase again.^37^ This suggests a nonlinear, inverted U-shaped relationship between hormone levels and GMV, as has been found in the dose-dependent E2 effects on hippocampal plasticity.^39^

Several limitations should be acknowledged with respect to the present findings and their possible interpretation. First, although some whole-brain and ROI-based analyses suggested anatomically plausible volume differences across the menstrual cycle, confirming the previous literature,^5^^,^^6^ these results did not survive correction for multiple comparisons. Thus, the exploratory results, which did not survive FDR correction, are only reported in the Supplement, and should be regarded as preliminary and hypothesis-generating as they are highly susceptible to false positives due to multiple comparisons. Second, although the sample size is adequate for ROI-focused longitudinal designs, it is modest for exploratory whole-brain analyses. This increases the uncertainty around smaller effects, emphasising the need for independent replication in larger cohorts to confirm any spatially distributed patterns suggested by the exploratory results. Third, the sample was intentionally homogeneous (young, cisgender, heterosexual, normal BMI, not in a relationship) because participants were drawn from a larger study on neural responses to body odour in fertile women and single men and women. While this homogeneity helps reduce hormonal and psychosocial confounds and increase internal validity, it limits generalisability to women with different ages or health profiles. In addition, the exclusion of the luteal phase, when P4 peaks and E2 remains elevated, potentially limits the generalisability of the midcycle hormone-brain associations. Fourth, the receptor density maps derived from the Allen Human Brain Atlas are based predominantly on male donors (five out of six brains), which represents a substantive limitation because sex differences in receptor distributions may influence the accuracy of colocalisation analyses. This sex imbalance should be considered when interpreting receptor-based mechanistic inferences.

Despite these limitations, the study has several strengths. This multimodal methodology of volumetric and surface-based morphometry increases the reliability and interpretability of the findings. Second, the inclusion of hormone assays and spatial receptor density mapping enhances the mechanistic understanding of the observed effects, linking macrostructural brain differences to potential underlying hormonal signalling pathways.

Taken together, our findings are in line with previous literature confirming that hormonal fluctuations across the menstrual cycle are associated with dynamic, region-specific changes. Although in previous studies E2-related changes have been reported in relation to regional grey matter changes, with a particular neural plasticity in the hippocampus and the amygdala, we found P4 to be a key modulator of both GMV and CT, likely due to several mechanistic insights. For instance, P4's neuroactive metabolite allopregnanolone enhances GABA-A receptor activity and promotes oligodendrocyte proliferation and myelination, processes more readily captured by MRI, whereas E2's effects are subtler, primarily synaptic, and less likely to appear in volumetric measures.^40^^,^^41^ Importantly, although BDI and PTSS scores differed between the phases, their lack of association with hormone levels may reflect the overall low symptom severity in this healthy sample, limited variability in questionnaire scores, or the influence of non-hormonal factors such as stress, sleep, or psychosocial context. Hormone-mood associations may be detectable primarily in individuals with higher symptom burden, such as those with PMS or PMDD. Finally, beyond macrostructural variation, the brain is organised into intrinsic functional connectivity networks that provide a complementary mechanistic framework for linking neural organisation to behaviour. Recent research has shown that patterns of intrinsic connectivity within large-scale networks are systematically related to cognitive, affective, and social functioning, thereby constraining individual differences in behaviour.^42^, ^43^, ^44^, ^45^ By integrating hormone-sensitive changes in brain morphology with alterations in intrinsic connectivity, future studies may more fully characterise how cyclical endocrine dynamics shape behaviour and mood across the female lifespan.

Although not all our results survived correction, converging evidence across volume, surface, and regression analyses, alongside receptor colocalisation, point to a biologically meaningful reorganisation of brain structure in response to endogenous hormonal variation. Importantly, these cyclical changes do not occur in isolation as P4-related neuroplasticity mirrors structural brain adaptations reported during other major reproductive transitions. Altogether, these findings suggest that the structure of the female brain reflects continuous dynamic hormonal influence across the lifespan, with P4 playing a central role. Elucidating these processes is critical not only for advancing our understanding of sex-specific neurodevelopment, but also for identifying periods of increased vulnerability to mood disturbances and other neuropsychiatric conditions in women.

## Contributors

Conceptualisation: SN, UH, NC. Data Curation: EL, MA. Formal Analyses: SN. Funding acquisition: UH, NC. Investigation: EL, MA. Methodology: SN. Project administration: UH, NC. Supervision: UH, NC. Visualisation: SN. Writing–original draft: SN Writing–review and editing: all authors. All authors (SN, EL, MA, UH, NC) confirm that they had full access to verify all underlying data in the study and accept responsibility to submit for publication. UH and NC contributed equally as last authors. All authors read and approved the final version of the manuscript.

## Data sharing statement

The data of this study are not publicly available due to privacy and ethical restrictions. Data to support the findings of this study are available upon reasonable request.

## Declaration of interests

The authors declare no conflicts of interest.
