# Supplementary material for: Progesterone-associated adjustments in brain structure during menstruation and the periovulatory phase—an MRI study
Source: eBioMedicine. 2026 Feb 20;125:106184. doi: 10.1016/j.ebiom.2026.106184 (PMC12945537; doi:10.1016/j.ebiom.2026.106184)
Supplement: Supplementary Figures [file mmc1.docx]

**Supplementary Information for:**

**Progesterone-associated adjustments in brain structure during menstruation and the periovulatory phase – an MRI study**

Susanne Nehls^1^, Elena Losse^1^, Maya Armin^1^, Ute Habel^1,3^ *, Natalia Chechko^1,2,3^ *

^1^Department of Psychiatry, Psychotherapy and Psychosomatics, Medical Faculty, RWTH Aachen, Aachen, Germany.

^2^Institute of Neuroscience and Medicine, Brain and Behavior (INM-7), Research Center Jülich, Jülich, Germany.

^3^Institute of Neuroscience and Medicine: JARA-Institute Brain Structure Function Relationship (INM-10), Research Center Jülich, Jülich, Germany.

***Shared last authorship: Habel and Chechko**

Corresponding author:

Susanne Nehls, snehls@ukaachen.de

**Results**

**Exploratory ROI analyses and whole-brain analyses**

At an exploratory uncorrected threshold, greater volume during the periovulatory phase (POV) compared to the menstrual phase (ME) emerged in the right hippocampus (p = .014, d = 0.76) and right amygdala (p = .026, d = 0.69).

Exploratory uncorrected analyses further identified greater volume during POV in the bilateral middle temporal gyri (right p = .001, d = 0.58, d =; left p = .013, d = 0.42), left cerebellum exterior (p = .015, d = 0.4), left central operculum (p = .026, d = 0.36), left fusiform gyrus (p = .028, d = 0.35), left inferior (p = .034, d = 0.34) and superior temporal gyri (p = .035, d = 0.33), right planum temporal (p = .036, d = 0.33), right inferior temporal gyrus (p = .041, d = 0.34), right inferior occipital gyrus (p = .042, d = 0.19), and right superior temporal gyrus (p = .045, d = 0.31) (Fig. S1).


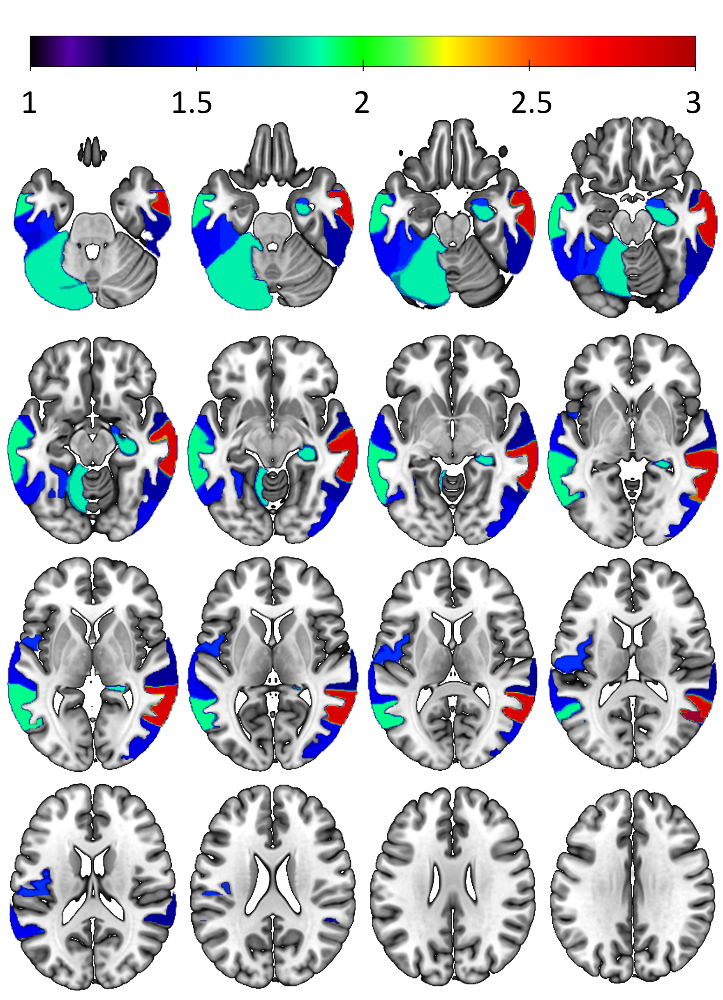


**Figure S1**. Regions-of-interest (ROI) analyses revealed greater volume during the periovulatory phase compared to menstruation. The differences did not survive FDR correction for multiple comparisons.

Exploratory uncorrected analyses further showed positive correlation between oestradiol (E2) and GMV in the left postcentral gyrus, supramarginal gyrus, and inferior parietal lobule (642 voxels, peak MNI coordinates -63, -28, 51; T = 5.78; p-uncorrected = .006; FDR corrected p = .068, Fig. 2A) and with progesterone (P4) and GMV inn the right fusiform and inferior temporal gyri (323 voxels, peak MNI 50, -56, -18, p-uncorrected = .039, Fig. 2B).


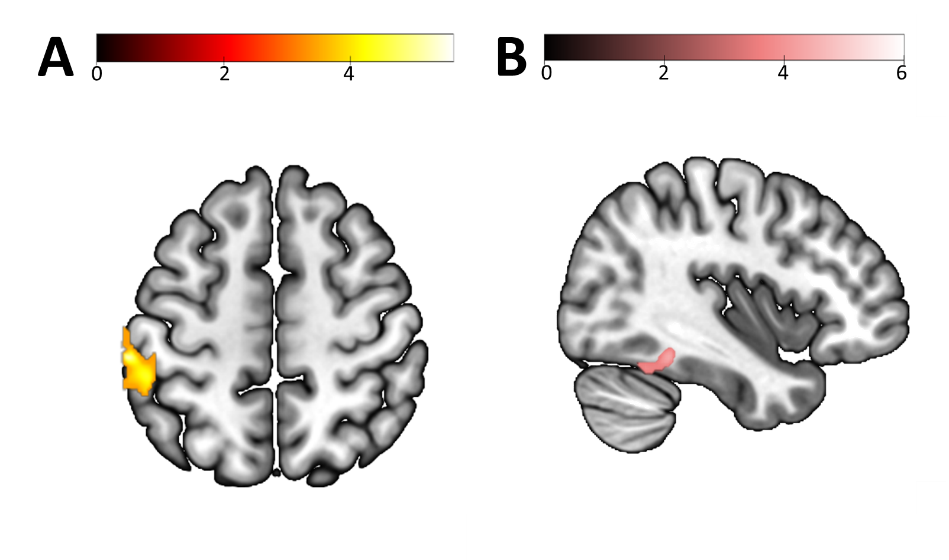


**Figure 2**. A) Uncorrected positive correlation between oestradiol levels and grey matter volume (GMV) in the supramarginal gyrus/inferior parietal lobule during the periovulatory phase (POV) B) Uncorrected positive correlation between progesterone levels and GMV in the fusiform gyrus during POV.
